# A Practical Approach to T-Cell Receptor Cloning and Expression

**DOI:** 10.1371/journal.pone.0027930

**Published:** 2011-11-21

**Authors:** Sébastien Wälchli, Geir Åge Løset, Shraddha Kumari, Jorunn Nergård Johansen, Weiwen Yang, Inger Sandlie, Johanna Olweus

**Affiliations:** 1 Department of Immunology, Institute for Cancer Research, Oslo University Hospital Radiumhospitalet, Oslo, Norway; 2 Department of Molecular Biosciences and Centre for Immune Regulation, University of Oslo, Oslo, Norway; 3 Department of Immunology, Oslo University Hospital Rikshospitalet and University of Oslo, Oslo, Norway; 4 Institute of Clinical Medicine, University of Oslo, Oslo, Norway; Federal University of São Paulo, Brazil

## Abstract

Although cloning and expression of T-cell Receptors (TcRs) has been performed for almost two decades, these procedures are still challenging. For example, the use of T-cell clones that have undergone limited expansion as starting material to limit the loss of interesting TcRs, must be weighed against the introduction of mutations by excess PCR cycles. The recent interest in using specific TcRs for cancer immunotherapy has, however, increased the demand for practical and robust methods to rapidly clone and express TcRs. Two main technologies for TcR cloning have emerged; the use of a set of primers specifically annealing to all known TcR variable domains, and 5′-RACE amplification. We here present an improved 5′-RACE protocol that represents a fast and reliable way to identify a TcR from 10^5^ cells only, making TcR cloning feasible without *a priori* knowledge of the variable domain sequence. We further present a detailed procedure for the subcloning of TcRα and β chains into an expression system. We show that a recombination-based cloning protocol facilitates simple and rapid transfer of the TcR transgene into different expression systems. The presented comprehensive method can be performed in any laboratory with standard equipment and with a limited amount of starting material. We finally exemplify the straightforwardness and reliability of our procedure by cloning and expressing several MART-1-specific TcRs and demonstrating their functionality.

## Introduction

Recently, T-cell receptors (TcRs) have gained interest as attractive immunotherapeutics in cancer [1], [2], [3], [4]. Proof-of-concept for the effectiveness of cancer-targeted TcRs in man was first demonstrated in malignant melanoma by Rosenberg *et al.* [5]. Here, a TcR isolated from a patient with an efficient anti-tumor response was transferred *in vitro* to T cells from patients that did not mount such responses. Regression of metastatic melanoma was observed in 2/15 TcR-treated patients, and the TcR-transduced T cells could be detected 12 months after treatment. These encouraging results have boosted the search for novel TcRs with utility in cancer-treatment. However, although the cloning of a murine [6] and a human [7] TcR was first performed almost three decades ago, this procedure remains challenging thus preventing rapid and widespread testing of novel TcRs for therapeutic use. Since the mRNA levels encoding TcR in a T cell are considerably lower compared with those encoding immunoglobulin in a B cell, cloning of the former represents a far bigger challenge when cell numbers are limited. Furthermore, as the risk of inducing errors in the sequence increases relative to the number of PCR cycles, it is advantageous to start with thousands rather than hundreds of cells. However, when expanding T-cell clones, a large fraction of the clones is invariably lost and the corresponding TcRs made unavailable for further testing. It is therefore desirable to have a method that allows the accurate discovery of the TcR sequence from clones that count less than a million T cells.

Among methods previously presented for TcR cloning, two have gained particular popularity due to their low cost and practicality. One method involves direct PCR amplification of T-cell cDNA, using a panel of primers annealing to all Variable regions of TcRs (V-region, more than 50 for TCRα and -β) [8], [9], [10]. This method has been used to perform single cell TcR cloning [11], [12]. The other is a 5′-RACE amplification. This elegant method was presented almost two decades ago [13] and was recently modified by other groups [14], [15], [16], [17]. By tailing the 5′-end of the total cDNA synthesized from a T-cell clone, TcRs could be amplified specifically without *a priori* knowledge of the identity of the V-region. This system was mainly restricted to sequence identification and provided valuable information of the TcR usage in a given cell line or clones. For many applications, it is desirable to express the TcR following sequence identification. Although the ectopic expression of TcR was accomplished 25 years ago [18] and the method has evolved [19], [20], [21], only a few examples of practical identification-expression interfaces have been proposed [16], [22].

In the present paper, we have improved the backbone of the 5′-RACE protocol for the identification of TcR sequences from a low number of T cells, but without an excessive number of PCR cycles to ensure accurate sequence identification. The TcR chains are then amplified using V-chain specific primers. By overlapping PCR, the TcR chains are linked using the picornavirus 2A peptide coding sequence to generate a TcR_2A transgene [23]. The somatic recombination at the CDR3 hypervariable domains, as well as the variability in the sequences of the V region, may generate restriction sites that complicate further subcloning. To avoid the need for tailor made primers accommodating restriction site requirements, we have developed a platform that exploits TOPO-cloning and recombination systems. This universal platform is a simple and rapid method to subclone any TcR_2A transgene into different expression systems. Here, we validate this method with MART-1 specific TcRs and show that they can be transferred to retroviral- or RNA-vector systems for subsequent expression and testing without extensive cloning design. The detailed protocol described herein can be established in any laboratory and represents a convenient method to isolate, identify, and study TcRs of interest.

## Results and Discussion

### Determination of TCRα and β chain identity

**Figure 1:**
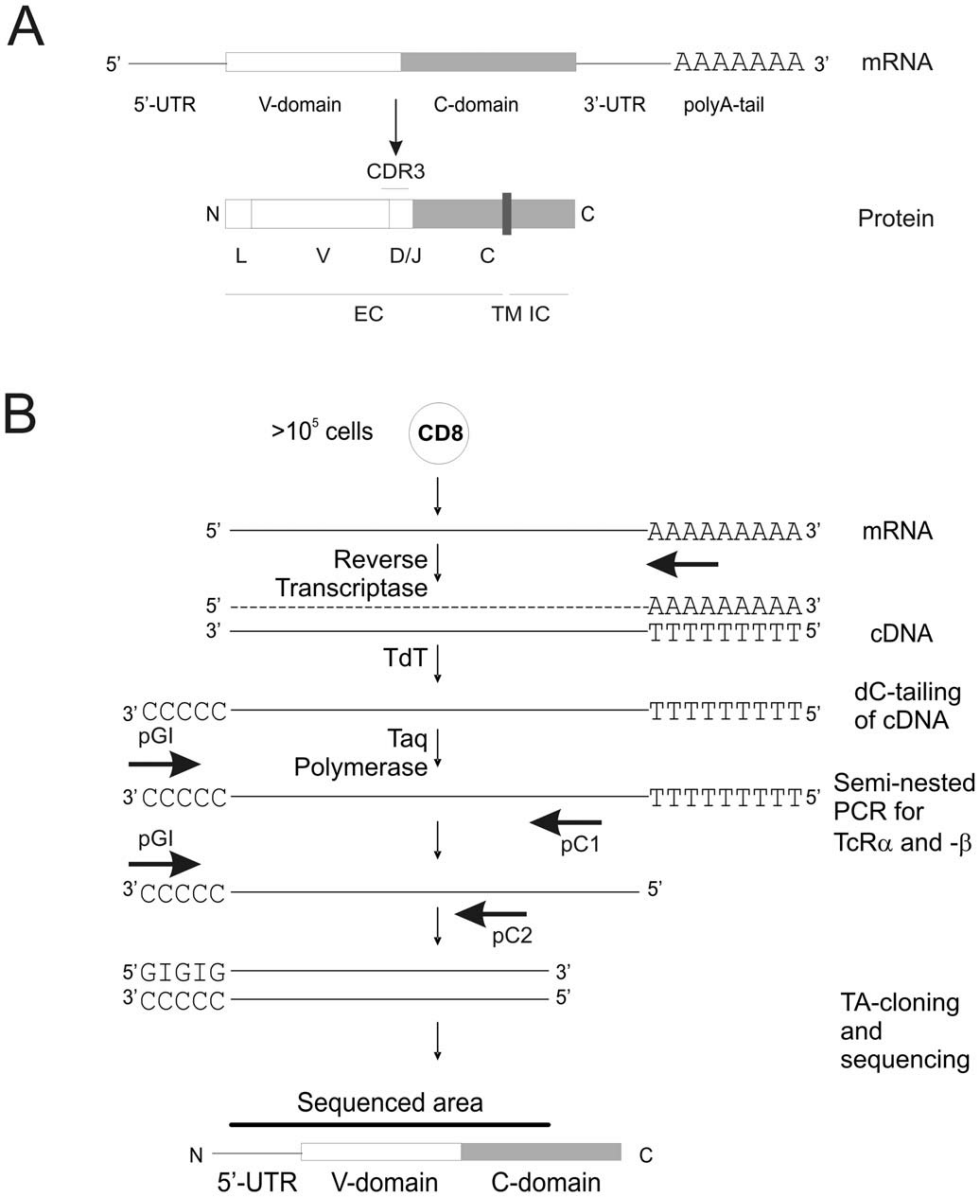
TcR structure and cloning strategy. (a) TcRα and β mRNA structures are similar: The 5′-situated Variable (V) domain encodes for the N-terminal part of the extracellular domain (EC) of the TcR. It starts with the signal sequence (L) upstream of the V-region. It ends at the recombination site with the D domain (only in TcRβ) and Joining domain (J), representing the hypervariable CDR3 domain. On the 3′-side of the messenger lies the Constant (C) region, which encodes for the carboxy-terminal part of the EC, the transmembrane region (TM) and the short intracellular domain (IC). (b) The 5′-RACE of the TcR is performed by reverse-transcription of mRNA into cDNA using an oligo dT primer. The cDNA is then polyC-tailed by TdT and this reaction is followed by two sequential amplifications using nested primers (pC1 and pC2) together with a polyC annealing primer (pGI). The final product is cloned into pTA and sequenced. The sequenced portion results in the full length V-domain and the CDR3 if the tailing has occurred on the 5′-UTR.

TcRα and β mRNA have similar structures, consisting of a constant 3′-end and a variable 5′-end. Upon translation they will generate Type 1 receptor proteins where the constant domain will cross the plasma membrane and provide the first half of the extracellular domain, followed by the variable domain with the hypervariable region CDR3 pointing at the top of the structure (Fig. 1a). The unknown identity of the variable region makes amplification of the receptor a challenge.

MART-1/Melan-A is overexpressed in melanoma and represents a well defined melanocyte marker used in diagnostics. A peptide derived from MART-1/Melan-A (MART-1 peptide_26–35_, ELAGIGILTV) that is presented by HLA-A2, has become a widely used immunotherapeutic target, and TcRs recognizing this complex have been isolated [24]. Our protocol was established with CD8+ T-cell clone derived from a cell line specific for the MART-1 peptide presented on HLA-A2. In a previous study, we sorted T cells reactive with HLA-A2/MART-1 multimers as single cells and expanded the resulting T-cell clones [25]. In the present study, these T-cell clones were utilized as the starting material for molecular cloning, using 10^5^ T cells from each clone. This reduces the loss of TcRs due to failure to adequately expand clones, while limiting the number of PCR-introduced errors in the subsequent cloning protocol. The RNA was prepared from fresh T cells (primary clones or SupT1 cell line, see below) or frozen pellets (see Materials and methods). The total RNA produced from these clones was checked for purity and, depending on the yield, as little as 300 ng was used to synthesize cDNA.

**Figure 2:**
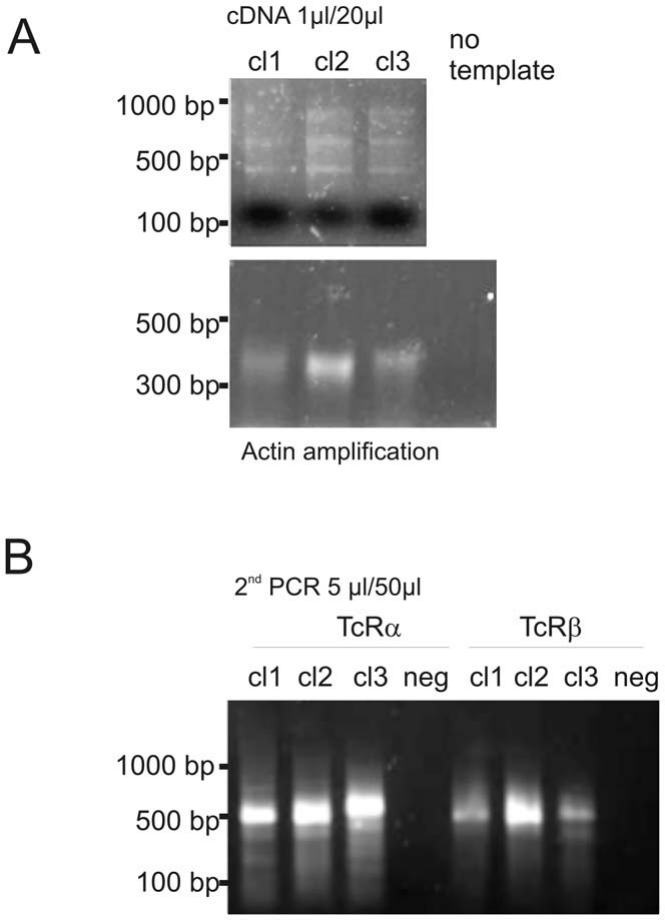
TcR cloning. (a) Three T-cell clones (1–3) were used to prepare total RNA. The mRNA was reverse-transcribed with oligo dT, as described in Fig. 1, and 1/20 of the cDNA was loaded on the gel to check reverse-transcription efficiency (upper gel). The same cDNAs were amplified using actin primers annealing to two different exons and 1/20 was loaded on the gel (lower gel). As a negative control, the same reaction was performed without template. (b) For the second PCR, 1/50 of the first PCR was amplified using a nested primer annealing to the 3′-end of the cDNA. When 1/10 of the PCR product was run on a 1.5% agarose gel, a smeared product could be observed.

As shown in Fig. 1b, the polyA RNA was reverse-transcribed using an oligodT primer. The quality of the resulting cDNA was checked either by actin amplification or direct loading of the cDNA onto an agarose gel (Fig. 2a). Since the identity of the TcR V was unknown, a polynucleotide anchor was added to the cDNA 3′-end of the V-domain side by tailing the purified cDNA with cytosines using the Terminal-deoxynucleotide-Transferase (TdT) in a 15 minute reaction (Fig. 1b). The tailed cDNA was then re-purified and amplified in a semi nested PCR: the 2 PCR reactions were performed using a modified poly-guanosine/inosine primer (pGI) together with specific primers for the constant domains of TcRα and β (pCa1/2 and pCb1/2, respectively; 1 for the first PCR and 2 for the second). Inosine preferentially replaces guanosine, but connects to cytosine with only two hydrogen bonds [26]. Consequently the pGI primer will have a decreased melting temperature during the PCR cycles. This represents an advantage over the use of poly-guanosine primers. The first PCR does not usually generate a visible gel fragment at the expected size (around 500 bp for both TcRα and β), but rather a weak smear (data not shown). This smear results from the different portions of the 5′-UTR that are reverse transcribed during cDNA synthesis as well as from non-specific amplicons. Indeed, direct cloning of this material provides only a few positive sequences for TcR and a large amount of contaminating sequences (non-TcR cDNA, data not shown). A second round of PCR using nested primers for the C domains (pC2) was thus performed to generate a product of higher specificity (Fig. 2b). Importantly, pCb-1 and pCb-2 are specific for both TcRβ isoforms since they were designed to anneal on overlapping stretches (Table 1 and data not shown). As expected, the second PCR generated a fragment of more than 450 bp in size. Smaller fragments will not contain the translation start site. Again a smear was observed on agarose gel, but it mainly consisted of specific bands as seen by subsequent sequencing. Since the PCR reactions were performed using Taq polymerase, the gel-purified amplicon could be cloned into a TA-opened plasmid. The expected plasmid insert should contain the full V-chain, the CDR3 and a piece of the C-domain. Separate bacteria colonies were picked from the same reaction. The sequences of 2 to 4 colonies were then compared in order to identify eventual PCR-generated errors. The identity of the sequenced insert was determined by comparing the sequence of interest against the public IMGT database ([27], http://imgt.cines.fr/). Therefore, even if the PCR generated errors present in all sequences, the identification of the correct V-gene would not be affected. Since identical TcR sequences were sometimes found in different original T-cell clones (sister clones), we here present 9 unique HLA-A2/MART-1 specific TcR sequences (Table 2). Some TcRα chains were not sequenced but were analyzed with specific primers recognizing TRAV12-2 only [25]. As reported in our previous study [25], TcRs specific for allogeneic HLA-A2 in complex with the MART-1 peptide showed a bias for TRAV12-2, in a similar way as TcRs recognizing this complex in autologous setting. [28], [29], [30]

**Table 1:** List of primer sequences and their use and/or specificity.

Primer name:	Sequence:	Comment:
pCa1	5′-AGTCAGATTTGTTGCTCCAGGCC-3′	reverse primer for 1st TCRα amplification
pCb1	5′-TTCACCCACCAGCTCAGCTCC-3′	reverse primer for 1st TCRβ amplification
pCa2	5′-ATACGCGTTCTCTCAGCTGGTACACGG-3′	reverse primer for 2nd TCRα amplification
pCb2	5′-ATACGCGTAGATCTCTGCTTCTGATGGC-3′	reverse primer for 2nd TCRβ amplification
pGI^a^	5′-CACCGGGIIGGGIIGGGII-3′	PolyGI with TOPO cloning sites
pCa-2A	5′-TGGTCCAGGGTTCTCTTCCACGTCGCCGGCCTGCTTCAGCAGGCTGAAGTTGGTGGCGCCGCTGCCTCTCTTGGCTCTGCTGGACCACAGCCGCAGCG-3′	Universal TcRα constant primer-2A peptide
pVb-2A^b^	5′-GCCGGCGACGTGGAAGAGAACCCTGGACCA-(VBspecific) -3′	Vβ primer fusing the 2A sequence
pVa^b^	5′-CACC-(Vaspecific) -3′	Vα specific primer
pC-STOPb1	5′-ATATCTCGAGTCAGAAATCCTTTCTCTTGAC-3′	TcRβ constant part Type 1 primer
pC-STOPb2	5′-ATATCTCGAGCTAGCCTCTGGAATCCTTTC-3′	TcRβ constant part Type 2 primer
pVa12-2^c^	5′-CACC*ATGATGAAATCCTTGAGAGT*-3′	Vα12-2 specific primer
pVb6-5^c^	5′-AGAGCCAAGAGAGCCACCAACTTCAGCCTGCTGAAGCAGGCCGGCGACGTGGAGGAGAACCCCGGTCCC*ATGAGCATCGGCCTCCTGTGC*-3′	Vβ6-5 specific primer
pVb28^c^	5′-AGAGCCAAGAGAGCCACCAACTTCAGCCTGCTGAAGCAGGCCGGCGACGTGGAGGAGAACCCCGGTCCC*ATGGGAATCAGGCTCCTGTG*-3′	Vβ28 specific primer
pActinF	5′-GCTCCGGCATGTGCAA-3′	Actin exon 2
pActinR	5′-AGGATCTTCATGAGGTAGT-3′	Actin exon 4

a I is desoxyinosine.

b These primers are designed when clone sequence is known.

c Sequence in italic is V-chain specific.

**Table 2:** TcR composition.

Origin		V domain composition			CDR3
SUPT-1	TcRα	TRAV1-1	TRAJ12	-	CAVPP # MDSSYKLIF
	TcRβ	TRBV9	TRBJ2-1	TRBD2	CASSVGGSLKQFF
B48	TcRα	TRAV41	TRAJ49	-	CAVNGTGNQFYF
	TcRβ	TRBV27	TRBJ1-5	TRBD1	CASSFGGLGQPQHF
B1	TcRα	TRAV12-2	TRAJ31	-	CAVSNARLMF
	TcRβ	TRBV2	TRBJ1-5	TRBD1	CASSDSGVGQPQHF
B11	TcRα	nd	nd	-	nd
	TcRβ	TRBV2	TRBJ1-6	TRBD1	CASISFQRQAPNSPLHF
**B44**	TcRα	TRAV12-2	TRAJ20	-	CAVKVYKLSF
	TcRβ	TRBV6-5	J1-2	TRBD1	CASSSPNRQGDYGYTF
B54	TcRα	TRAV4	TRAJ40	-	CLVGSASGTYKYIF
	TcRβ	TRBV4-1	TRBJ1-5	TRBD1	CASSQDPGLHQPQHF
**B60**	TcRα	TRAV12-2	TRAJ23-1	-	CAAQGGKLIF
	TcRβ	TRBV28	TRBJ1-3	-	CASSPVGLSGNTIYF
B36	TcRα	TRAV12	nd	-	nd
	TcRβ	TRBV6-2	TRBJ1-1	TRBD1	CASSYLGQPTLNSANTGELFF
B9	TcRα	TRAV12	nd	-	nd
	TcRβ	TRBV4-2	TRBJ1-1	TRBD1	CASSPGPLLTEAFF

# : frameshift.

nd: not determined.

Our protocol was also tested on a commonly used T-cell line, SupT1. SupT1 is derived from a lymphoma with a chromosomal inversion that renders it TcRα negative [31], but is often wrongly described as TcRαβ negative. We confirmed other reports claiming that these cells express a functional TcRβ [32], but also found that they express TcRα mRNA encoding a non-functional receptor (Table 2). As discussed below and by others [11], this type of mRNA is often found during T-cell analysis. Importantly, as seen for SupT1, 3 out of 9 MART-1 specific colonies possessed a non-functional TcRα (not shown in Table 2) in agreement with previous observations [11], [33]. These non-functional TcRα chains can lead to misinterpretations in the TcR V-region analysis performed by PCR profile analysis, unless sequencing is performed. The TcRβ were also sequenced and did not show any strong bias in the Vβ usage (Table 2). Taken together we present a straightforward protocol to analyze the sequence of TcRs of interest from a low number of cells.

### Subcloning of TcRαβ into a flexible expression system

An expression construct was designed by fusing TcRα and β chains with a 2A sequence of picornavirus, a strategy described by others [19], [23], [34], [35], [36]. Briefly, 2A refers to a sequence that promotes ribosomal skipping, thus allowing the release of the upstream protein and translation of the downstream gene, which is of interest in multicistronic expression vectors. Since somatic recombinations at the hypervariable regions, and V domain variations [37] may generate novel restriction sites that potentially interfere with enzyme-based cloning, we exploited a recombination technology that permits the design of real universal primers for any TcR V-domain (Table 1).

**Figure 3:**
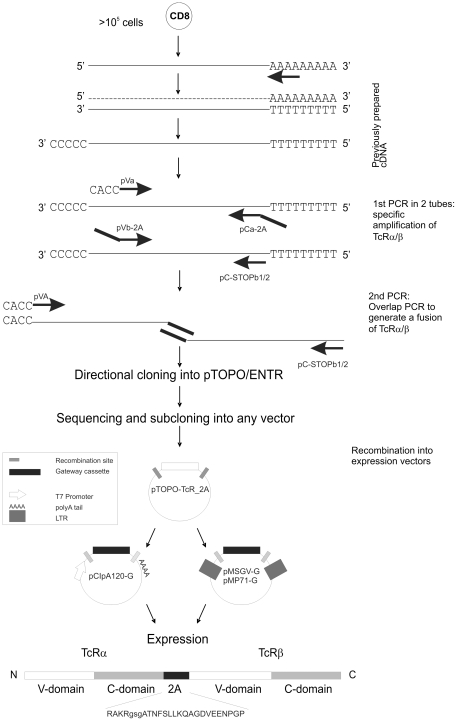
TcR_2A cloning strategy. Full length TcRα and β were re-amplified from cDNA using specific primers in 2 separate PCR reactions: The TcRα STOP codon was removed and a 2A coding sequence is fused to its 3′-end (pCa-2A). At the 5′-end of the TcRα, a CACC sequence was fused to the ATG to generate a Kozak sequence and also to orient the fragment for TOPO cloning (pVa). TcRβ was amplified using a forward primer that fused the 2A sequence in frame with its ATG (pVb-2A). The reverse primer (pC-STOPb1/2) contains the STOP codon. Importantly, the forward primers (Va and Vb specific) were designed to anneal the signal sequence (L) to the V region. The second PCR was overlapping and run using gel purified fragments of the two TcR chains and the external primers (pVa and pC-STOPb1/2). The amplicon was finally cloned into a TOPO-directional vector in order to generate pTOPO-TcR_2A. This vector possesses recombination sites that can be used for recombination into expression vectors.

As shown in Fig. 3, the polyC-cDNA template generated for sequence determination was also used for expression construct amplification. The 2A sequence was fused to the TcRα and β chains by overlapping PCR, using a high fidelity polymerase. The Ca-2A primer is universal; it contains the complete 2A coding sequence and removes the natural STOP of the TcRα. The resulting 2A sequence is given in Fig. 3 and has some additional features consisting of a furin site followed by an SGSG linker upstream of the 2A peptide. The furin site is thought to remove the 2A peptide remaining on the TcRα chain, and the short amino acid extension (SGSG) has been shown to improve the production of functional TcRs compared to the 2A sequence only, as described in [34]. The 2A-Vb is unique and can be smaller than the Ca-2A: an overlap of 27 nucleotides on the 2A side is sufficient to generate a fusion product. The Va-primer is unique, and should contain a CACC sequence before the ATG (5′-end) to orient it for the TOPO cloning. Finally, the Cb primers are also universal, but specific for either TcRβ of the C1 or C2 types. A restriction site (here *Xho*I) can be added to these primers in order to verify the cloning (Table 1). The final product is subcloned into a directional entry-vector (pTOPO-ENTR) yielding pTOPO-TcRα-2A-TcRβ (pTOPO-TcR_2A).

### Expression of TcR using different vectors

After sequencing of the pTOPO-TcR_2A, the insert can be recombined into any type of expression vector without the need for additional sequencing. Since TcR expression is mainly performed by mRNA electroporation or retroviral transduction, we converted different expression plasmids into Gateway™ destination vectors by adding a recombination cassette (see Materials and Methods). We selected a validated mRNA expression vector, pCIpA120 [38], and two retroviral vectors, pMSGV [17] and pMP71 [39]. These modified vectors, named pCIpA120-G, pMSGV-G and pMP71-G, respectively, are destination vectors with cassettes that can be replaced by any pENTR-containing insert in a one-step recombination.

**Figure 4:**
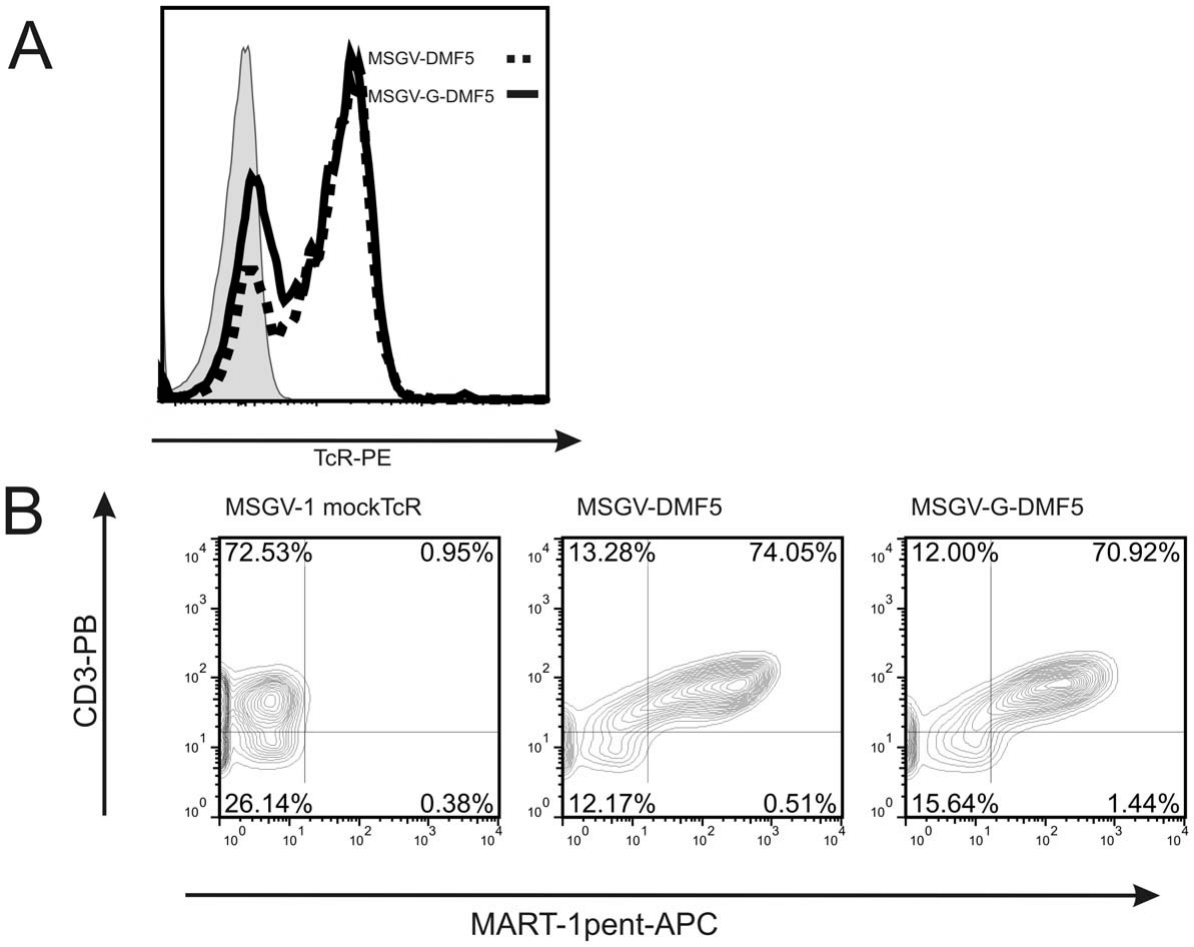
Validation of the recombination-system. (a) SupT1 cells were transduced with the indicated retroviral supernatant or mock-transduced (grey) by spinoculation and cultured for 2 days. They were then washed and stained with anti-TcR-PE and analyzed by flowcytometry. DMF5 inserted into MSGV or MSGV-G vectors resulted in comparable expression. (b) Three days post-transduction, SupT1 were co-stained with anti-CD3 PB and HLA-A2/MART-1 multimer-APC. SupT1 transduced with an irrelevant TcR were used as a multimer negative control. The percentage of cells is shown in each quadrant.

We first wanted to investigate if the Gateway™ cassette modification potentially interfered with protein production. We therefore subcloned a validated MART-1-specific TcR, DMF5 [40] into a pENTR vector and recombined it into the converted pMSGV-G. SupT1 cells were transduced with the original pMSGV-DMF5 construct in parallel with the Gateway™-construct, pMSGV-G-DMF5. TcR expression efficiencies were then compared by anti-TcRαβ staining and were shown to be equal with both backbones (Fig. 4a). Co-staining of HLA-A2/MART-1 multimer and anti-CD3 further demonstrated similar levels of binding and expression. From these data, we concluded that the addition of recombination sites did not interfere with TcR expression (Fig. 4b).

**Figure 5:**
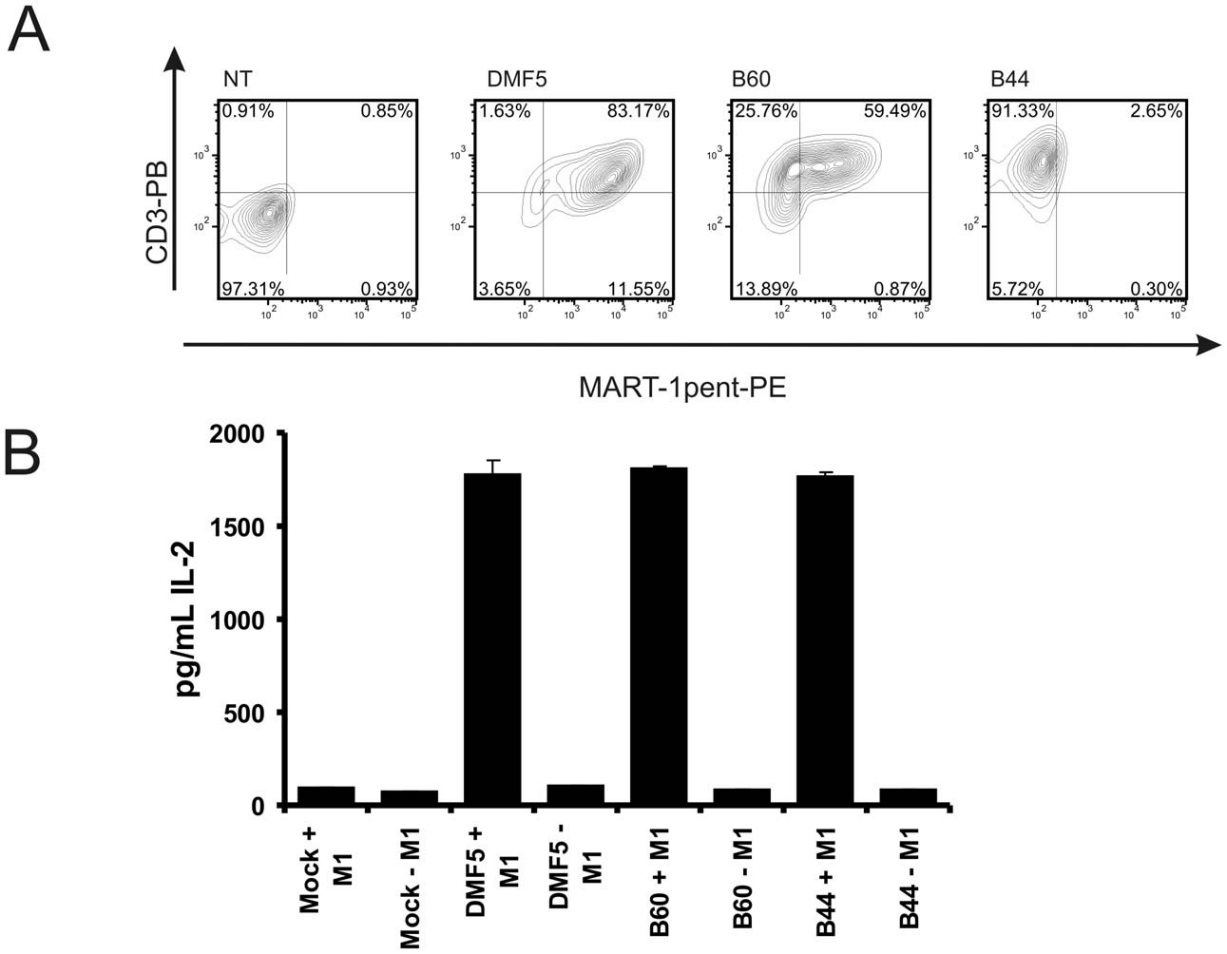
Retroviral delivery of MART-1 specific TcRs. (a) B44, B60 and DMF5 TcR_2A were expressed in the pMP71-G vector and transduced into SupT1 cells. After 3 days, cells were co-stained with anti-CD3 PB and HLA-A2/MART-1 multimer-PE. (b) Jurkat cells were transduced with the same constructs as in (a) or with a GFP pMP71-vector (Mock) and incubated for 24 hours with T2 cells loaded with or without MART-1 peptide (10 µM final concentration). IL-2 release was monitored by ELISA assay, and plotted as pg of IL-2 per mL of medium. Each bar represents the mean values of duplicates. Similar results were observed in two separate experiments.

Two in-house HLA-A2/MART-1 allo-restricted TcRs (B44 and B60, bold in Table 2) were next compared to the DMF5 TcR. All constructs were sub-cloned into the pMP71-G vector and transduced into SupT1 cells to measure their expression and binding efficiencies. As shown in Fig. 5a, all TcRs were expressed (CD3 staining). The binding capacities were however different; B60 seemed to require higher levels of TcRs on the surface for multimer binding than DMF5, since cells staining with similar levels of CD3 showed higher levels of multimer binding when transduced with DMF5. Moreover, B44 was not able to bind HLA-A2/MART-1 multimers, despite the fact that the original clone was selected for its pentamer-binding ability [25]. The use of Dasatinib, a SRC/ABL inhibitor shown to increase multimer signal by preventing TcR internalization [41], did not restore B44 binding (data not shown). In contrast to SupT1 cells, Jurkat cells can secrete IL-2 upon activation. Thus, by measuring IL-2 release it was possible to monitor TcR activity. Importantly, we have observed that TcR expression in our strain of Jurkat cells was highly increased when the MP71 retroviral vector was used, whereas SupT1 cells were not sensitive to the type of vector used (data not shown). We therefore transduced Jurkat cells with the MP71-constructs or a control construct (pMP-71-GFP). Seven days after transduction, MART-1 specific TcR-expressing Jurkat were incubated with HLA-A2 positive T2 cells, loaded or not with MART-1 peptide. Detection of IL-2 release was performed by ELISA (Fig. 5b). As shown, the three TcR constructs had a similar capacity to stimulate Jurkat cells in a peptide dependent manner, whereas Jurkat cells alone did not release IL-2 in any conditions.

**Figure 6:**
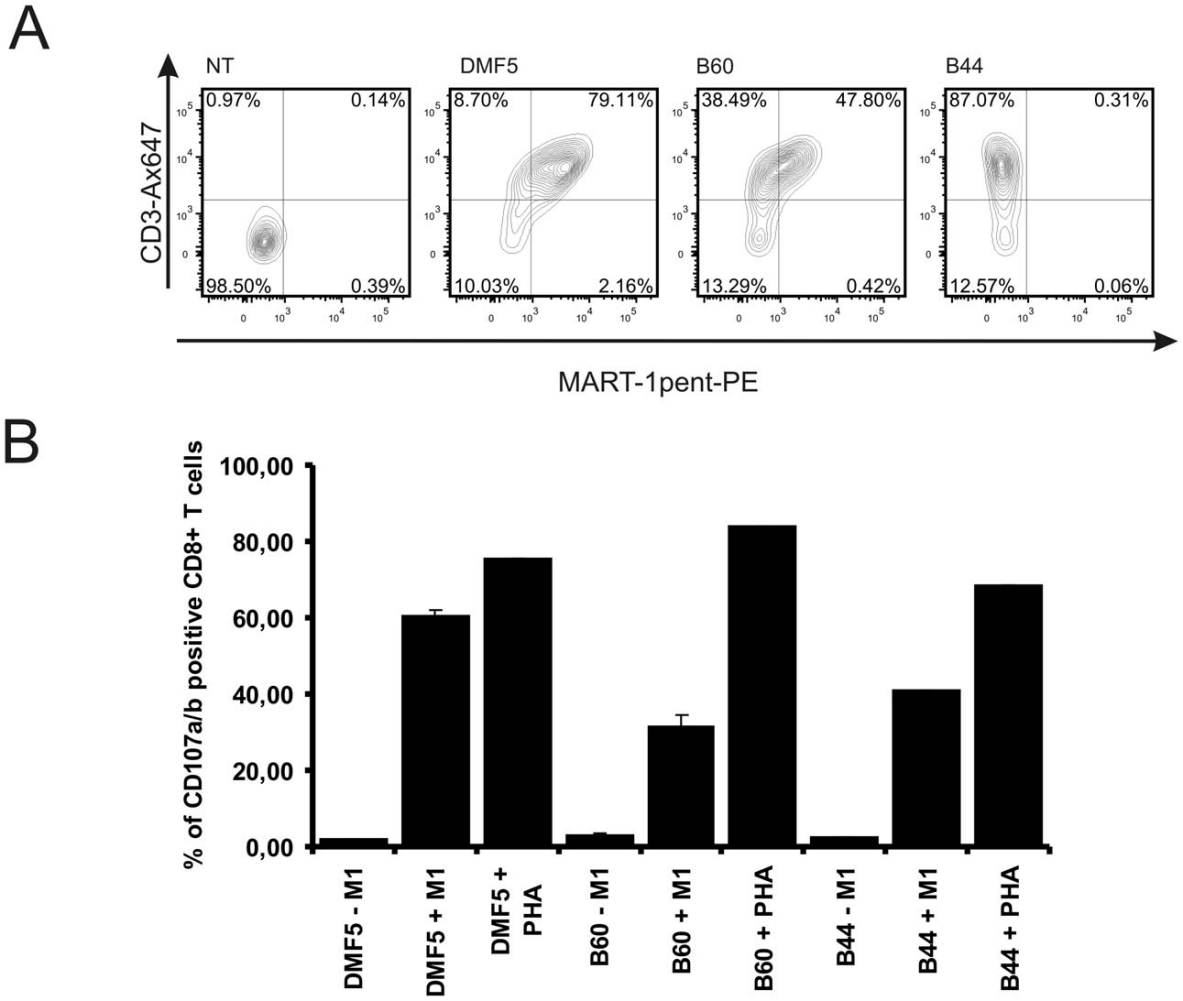
Electroporation of mRNA encoding MART-1 specific TcRs. (a) SupT1 were electroporated with or without 20 µg mRNA encoding the indicated TcR_2A. Twelve hours later, the cells were stained with HLA-A2/MART-1 multimer-PE and anti-CD3 Alexa Fluor 647. (b) Human PBMC were electroporated with 20 µg mRNA (same constructs as in (a)) and cultured for 4.5 hours. Following the addition of T2 cells pre-loaded or not (grey) with MART-1 peptide (10 µM final concentration), the cells were co-incubated for an additional 5 hours in the presence of anti-CD107a/b Alexa Fluor 647 antibodies, monensin and brefeldin A. Prior to analysis, cells were stained with anti-CD8 PE. The percentage of CD107a/b positive cells from the CD8 positive population is plotted. PHA was used to control for similar maximal degranulation levels regardless of the mRNA used for electroporation. Each bar represents the mean values of duplicates.

Another attractive method to express TcRs is by mRNA electroporation. We prepared mRNA *in vitro* using the pCIpA120-G modified vector. Again, SupT1 were used to test the TcR expression upon mRNA electroporation. The cells efficiently expressed the three TcRs after 12 hours as measured by anti-CD3 staining (Fig. 6a). As seen with the retroviral transduction (Fig. 5a), B44 did not react with the multimer whereas the other two constructs were positive. Finally, PBMC were electroporated with the mRNA of the three TcRs, and TcR activity was monitored 4.5 hours later. Redirected PBMC were incubated with T2 cells loaded or not with MART-1 peptide. To evaluate activation of CD8+ T-cells, degranulation was measured as expression of CD107a/b [42]. As shown in Fig. 6b, CD8+ T cells expressing DMF5 TcR were efficiently stimulated with peptide-loaded T2 cells. Although T cells expressing either of the two allogeneic TcRs were also specifically stimulated, they were less efficient than DMF5-expressing cells. As a control, all transduced cells were also incubated with 1 µg/mL phytohemaglutinin (PHA) to verify that the electroporation was not affecting the degranulation capacity of the cells. Taken together, the results show a discrepancy between multimer staining and functional avidities, as B44, which did not stain with multimers, induced higher functional responses than B60, which was positive in multimer staining.

This phenomenon has been previously reported by the group of Schendel [43]. They observed that peripheral blood lymphocytes transduced with two different TcRs showing virtually identical HLA-multimer staining frequencies and intensities, had a 100-fold difference in the sensitivity for peptide recognition, as measured in functional assays [43]. They concluded that there is no significant correlation between multimer analysis and functional avidity. Another explanation can be that DMF5 might be more efficient at recruiting CD3. Indeed Ahmadi *et al.* [44] recently showed that increased levels of CD3 by retroviral transduction of T cells increases the expression and the signaling of a redirecting TcR. In this report they showed that a TcR acquired the capacity to bind its cognate pentamer only if CD3 was co-expressed. One can speculate that TcRs B44 and B60 would gain activity with extra CD3. Moreover, DMF5 is known to be CD8 independent [40], whereas B60 is CD8 dependent for multimer staining (data not shown).

Although the B44-redirected T cells were not able to bind multimers, the original clone T-cell clone B44 was selected for its multimer binding capacity [25]. The TcR level of the original clone was likely higher than following ectopic expression, and multimer staining may require higher levels of TcRs. Nevertheless, the levels obtained by retroviral transduction or mRNA electroporation were sufficient to trigger a specific response in the functional assays.

Finally, a factor to take into account when comparing different TcRs in a transient transfection setup is their stability. We observed that B60 mRNA-transduced T cells showed a reduced signaling ability upon stimulation by MART-1 peptide loaded T2 cells at 10 hours, compared to 4.5 hours, after electroporation (Fig. 6b and data not shown), whereas DMF5 and B44 were more stable (data not shown). It is therefore tempting to speculate that B60 has a faster turn-over than DMF5 and B44.

These data suggest that multimer staining should always be combined with a functional assay to confirm the expression and specific activity of a receptor of interest.

### Conclusion

The molecular cloning of a heterodimeric receptor is a complex task. An improved method to reliably clone and express TcR from a limited number of T cells is presented. The TcR analysis protocol can easily be run in any laboratory without special equipment. Since the tailing is performed on cDNA, the time required to work with RNA is limited, reducing the problem of RNA degradation. By using the last generation of reverse transcriptase, and selected kits (see Materials and Methods), we obtained an optimal production of cDNA. Furthermore, with this procedure, there is no need to acquire a library of Vα and Vβ-specific primers, as the few primers listed in Table 2 suffice to identify any TcR.

Following the identification of the TcRα and β identities, the expression strategy was to use a 2A linker between the 2 chains [23]. We have further developed this technique by integrating the TcR_2A constructs in a recombination system (Gateway™ system). The main benefit is the universality of the primers used and the independence of restriction sites, which might easily be generated in hyper-variable regions. For versatile expression applications, we demonstrate the advantage of a recombination-based system that allows rapid switching of the TcR construct between vectors, as illustrated by production of the same TcR by use of an RNA-based system as well as a retroviral vector.

## Materials and Methods

### Antibodies and cell lines

The following antibodies were used: anti-humanTcR (T10B9.1A-31) Phycoerythrin (PE), anti-CD3 (OKT-3) Pacific Blue (PB), anti-CD8 (RPA-T8) PE, anti-CD107a (H4A3) Alexa Fluor 647, anti-CD107b (H4B4) Alexa Fluor 647 (BD Biosciences, Erembodegem, Belgium), HLA-A2/MART-1 multimer-PE or –APC were either from ProImmune Ltd. (Oxford, UK) or prepared in-house. Anti-CD3 Alexa Fluor 647 was labeled in-house. For functional assay, Brefeldin A and Monensin A were from Sigma-Aldrich (St. Louis, MO, USA.) MART-1 peptide_26–35_ (ELAGIGILTV) was from ProImmune.

SupT1 cells were a kind gift from M. Pule (University College London, UK) and Jurkat cells constitutively expressing CD8 were a kind gift from M. Nishimura (Medical University of South Carolina, Charleston, SC, USA). Packaging Hek-Platinium (Hek-P) cells were from Cell Biolabs (San Diego, CA, USA). SupT1 and Jurkat cells were grown in RPMI-1640 (PAA, Paschung, Austria), whereas Hek-P were grown in DMEM (PAA). All mediums were supplemented with heat-inactivated 10% fetal calf serum (FCS, HyClone, Logan, UT, USA) and 100 U/mL penicillin/streptomycin (PAA). PBMC were isolated and maintained as described in [25].

### TcR cloning

T-cell clones counting at least 10^5^ cells were stored as pellet at −80°C prior to RNA isolation (Fig. 1b). RNA was prepared using Absolutely RNA Miniprep kit (Stratagene, La Jolla, CA, USA), following the manufacturer's recommendations. The RNA was eluted in 50 µL elution buffer (pre-warmed at 65°C). The yield varied from 10–200 ng/µL. The RNA was checked for quality and quantity by Agilent 2100 Bioanalyzer (Agilent Technologies, Santa Clara, California) in the core facilities of Oslo University Hospital Radiumhospitalet, Oslo, Norway. Preparation of cDNA was performed using the maximum amount of RNA (from 100 ng). SuperScript™ III Reverse Transcriptase (Invitrogen, Carlsbad, CA, USA) was used following instructions, together with oligodT primers (Invitrogen) and the addition of RNAsin® (Stratagene) in the mix. The reaction was left for 1 hour at 50°C and RNAsin was inactivated at 60°C for 15 min. The mix was then RNAseH treated (New England Biolabs, Ipswich, MA, USA) at 37°C for 20 min. The cDNA was then precipitated: 22 µL cDNA+ 0.5 µL Glycogen (Fermentas, St. Leon-Rot, Germany) + 1/10 V sodium acetate 3 M pH 5.6+2.5 V Et-OH 100%. This mix was incubated at −20°C for 20 min, spun down for 10 min at 10,000×g at 4°C, ethanol-70% washed and the dry pellet resuspended in 11 µL dH2O. The 3′ terminal dC tailing of the cDNA was done in the following mix: 10 µL of the cDNA was heated at 95°C for 1 min and chilled on ice before tailing. TdT enzyme and buffer (Roche, Basel, Switzerland) were mixed with dCTP and cDNA in a final Volume of 20 µL and incubated at 37°C for 15 min. This mix was again glycogen/sodium acetate precipitated as previously described and re-suspended in 24 µL dH_2_O.

V chain amplification was performed in two steps (Fig. 1b). All PCR reactions were performed with Titanium Polymerase (Clontech, Saint-Germain-en-Laye, France) and the primers ordered HPLC-grade (Eurofins MWG Operon, Ebersberg, Germany). The first PCR was performed with the following primers: pCa1 and pGI (TcRα) and pCb1 and pGI (TcRβ) under these conditions: 25 cycles (1′ 94°C, 1′ 53°C and 1′ 68°C). The second PCR was nested. It was performed with the following primers: pCa2 and pGI (TcRα) and pCb1 and pGI (TcRβ) under these conditions: 20 cycles (1′ 94°C, 1′ 53°C and 30′ 68°C).

The amplicons were gel purified with E.Z.N.A. Cycle-pure Kit (Omega Bio-Tek Inc., Norcross, GA, USA) and finally cloned into pGEM vector using TA cloning technology (Promega, Madison, WI, USA). Clonings from low amounts of starting material were selected with blue/white screen. Colonies were picked, *Eco*RI (New England Biolabs) cut to monitor insert size and sent for sequencing (Eurofins MWG Operon) using M13rev(−29) and M13uni(−21) primers provided by the vendor. Sequences were analyzed online and named according to IMGT database (http://imgt.cines.fr/).

### TcR_2A construct cloning

All PCRs were performed using Pfx polymerase (Invitrogen). A first PCR was run to separately amplify TcRα and β (Fig. 3) using pVa and pCa-2A (TcRα) and pVb-2A with pC-STOPb1/2 (TcRβ, Table 1) with the following conditions: 25 cycles (1′ 94°C, 1′ 53°C and 1′ 68°C). The amplicons were run on an agarose gel and bands were purified (Omega Bio-Tek Inc.) for the second PCR. An equimolar amount (estimated on gel) of the fragments were mixed together with pVa and pC-STOPb1/2 and the PCR was run under these conditions: 20 cycles (1′ 94°C, 1′ 53°C and 2′ 68°C). The product was then cloned into pTOPO-ENTR vector (Invitrogen). DNA was extracted from 4 different Kanamycin resistant colonies and sequenced (Eurofins MWG Operon), using pTOPO primers (M13rev(−29) and M13uni(−21)) and a pair of internal primers: pCb2 and pCa2. Positive constructs were subcloned by recombination in any destination vector (see text), following manufacturer's instructions.

The construct pMSGV-DMF5_2A was a kind gift from R. Morgan (NIH, Bethesda, USA).

### Conversion to Destination vector

pDEST-51 (Invitrogen) was used as a template for amplification of the Gateway™ cassette and ligated into pGEM by TA cloning. Ten ng of pDEST-51 was amplified with the following primers: 5′-ATA TAC AAT TGA GGG TTA GGG ATA GGC TTA C-3′ and 5′-ATA TCT CGA GTA ATA CGA CTC ACT ATA GGG-3′, using Pfx for 25 cycles (1′ 94°C, 1′ 53°C and 2′ 68°C). TA-overhang was added using 7 µL clean PCR product together with 1 µL Taq buffer 10×(100 mM Tris, 15 mM MgCl_2_, 500 mM KCl set at pH 8.3), 1 µL Taq and 1 µL dATP (2 mM) at 70°C for 30 min and ligated 1 hr at room temperature (RT) with T4 ligase (Promega), dialyzed against dH_2_O and 2 µL of this was electroporated in DB3.1 (resistant to the suicide gene, *ccd*B) on ampicilin/chlorophenicol plate. This construct was sequenced and used to further subclone the cassette. The conversion of pMSGV (a kind gift from R. Morgan, NIH, Bethesda, USA) was done by *Xho*I/*Eco*RI or *Mun*I digestion. The conversion of pMP71 (a kind gift from W. Uckert, Berlin, Germany), was performed by blunting a *Not*I site in pMP71 and inserting the gateway cassette by blunt and *Eco*RI or *Mun*I digestion. The cloning of pCIpA120-G was perfomed using the same enzymes as for pMSGV.

### Retroviral particle preparation and transduction

Supernatants were prepared in Hek-P cells. Briefly, 1.2×10^6^ Hek-P were plated in 6 cm plates. Transfection was performed using Fugene-6 (Roche) with a mix of DNA including the retroviral packaging vectors and the expression vector to an equimolar ratio. After 24 hours, cells were transferred to a 32°C incubator and medium was replaced with 1% FCS containing DMEM. Supernatants were harvested after 24 and 48 hours incubation.

Transduction of SupT1 and Jurkat cells was done as follows: 2.5×10^5^ cells in a 12-well non-treated culture plate pre-coated with 0.5 mL/well Retronectin (20 µg/mL, Takara Bio. Inc., Shiga, Japan) were spinoculated with 1 mL of retroviral supernatant at 900×g for 60 min at 32°C. After 2 days, cells were harvested with PBS-EDTA 0.5 mM and grown in RPMI-10%FCS.

### mRNA preparation and electroporation

DNA plasmid was used as a template, it was opened with *Mfe*I (New England Biolabs), which lies downstream of the polyA tail [38]. Small scale mRNA was prepared using the mMESSAGE mMACHINE® T7 kit (Ambion, Austin, TX, USA) following manufacturer's instructions.

### TcR expression analysis

Around 10^5^ cells were resuspended in a solution containing the indicated antibody or multimer at 10 µg/mL for 20 min at RT. Cells were washed twice with Flow buffer (PBS, 2% FCS, 0.5 mM EDTA), and analyzed using LSR II flow cytometer (BD Biosciences). Figures were prepared using FlowJo software (Tree Star Inc., Ashland, OR, USA).

Functional assays for the measurement of degranulation (CD107a/b) were performed as described in [25]. Expanded T cells were electroporated at 7.5 µg mRNA/1 million cells in BTX ECM 830 (BTX, Harvard Apparatus, Holliston, MA, USA) square-wave electroporator in 2 mm cuvettes at 250 V for 2 ms. Cells were immediately grown at 1 million/mL CellGro DC medium (CellGenix, Freiburg, Germany) containing 5% human serum and 100 U IL-2 (Peprotech EC Ltd., London, United Kingdom)/mL pre-warmed at 37°C. Electroporated T cells were subsequently incubated at 37°C for 3 hr. After that the medium was changed and IL-2 removed for 1.5 hours before addition of the target cells.

IL-2 ELISA was performed following manufacturer's procedure (R&D Systems, Abingdon, United Kingdom).
